# Factors associated with increased lactate levels in cardiac surgeries: scoping review

**DOI:** 10.1590/0034-7167-2023-0117

**Published:** 2024-03-15

**Authors:** Fernanda de Castro Teixeira, Thatiane Evelyn de Lima Fernandes, Karena Cristina da Silva Leal, Katia Regina Barros Ribeiro, Daniele Vieira Dantas, Rodrigo Assis Neves Dantas

**Affiliations:** I Universidade Federal do Rio Grande do Norte. Natal, Rio Grande do Norte, Brazil

**Keywords:** Cardiac Surgery, Lactic Acid, Mortality, Post-Operative Period, Extracorporeal Circulation, Cirugía Cardíaca, Ácido Láctico, Mortalidad, Periodo Posoperatorio, Circulación Extracorporea, Cirurgia Cardíaca, Ácido Láctico, Mortalidade, Período Pós-Operatório, Circulação Extracorpórea

## Abstract

**Objectives:**

to map the factors associated with increased lactate levels in the postoperative period of cardiac surgery using extracorporeal circulation.

**Methods:**

this is a scoping review carried out in December 2022, across ten data sources. It was prepared in accordance with the recommendations of the Joanna Briggs Institute and the Preferred Reporting Items for Systematic Reviews and Meta Analyses Extension for Scoping Reviews checklist.

**Results:**

the most recurrent findings in studies regarding the factors responsible for the increase in lactate were: tissue hypoperfusion, cardiopulmonary bypass time and use of vasoactive drugs. In 95% of studies, increased lactate was related to increased patient mortality.

**Conclusions:**

discussing the causes of possible complications in cardiac surgery patients is important for preparing the team and preventing complications, in addition to ensuring quality recovery.

## RESUMO

**Objetivos:**

mapear os fatores associados à elevação do nível de lactato no pós-operatório de cirurgia cardíaca com uso de circulação extracorpórea.

**Métodos:**

trata-se de uma revisão de escopo realizada em dezembro de 2022, em dez fontes de dados. Foi elaborada conforme as recomendações do Instituto Joanna Briggs e do *checklist Preferred Reporting Items for Systematic Reviews and Meta-Analyses Extension for Scoping Reviews*.

**Resultados:**

os achados mais recorrentes nos estudos a respeito dos fatores responsáveis pelo aumento do lactato foram: hipoperfusão tecidual, tempo de circulação extracorpórea e uso de fármacos vasoativos. Em 95% dos estudos, o aumento do lactato relacionou-se ao aumento da mortalidade dos pacientes.

**Conclusões:**

discutir sobre as causas de possíveis complicações em pacientes de cirurgia cardíaca faz-se importante para o preparo da equipe e prevenção de intercorrências, além de garantir recuperação de qualidade.

## RESUMEN

**Objetivos:**

mapear los factores relacionados a la elevación del nivel de lactato en el posoperatorio de cirugía cardíaca con uso de circulación extracorporea.

**Métodos:**

se trata de una revisión de ámbito realizada en diciembre de 2022, en diez fuentes de datos. Fue elaborada conforme las recomendaciones del Instituto Joanna Briggs y del *checklist Preferred Reporting Items for Systematic Reviews and Meta-Analyses Extension for Scoping Reviews*.

**Resultados:**

los hallados más recurrentes en los estudios sobre los factores responsables por el aumento del lactato fueron: hipoperfusión tisular, tiempo de circulación extracorporea y uso de fármacos vasoactivos. En 95% de los estudios, el aumento del lactato se relacionó al aumento de la mortalidad de los pacientes.

**Conclusiones:**

discutir sobre las causas de posibles complicaciones en pacientes de cirugía cardíaca se hace importante para el preparo del equipo y prevención de intercurrencias, además garantizar recuperación de calidad.

## INTRODUCTION

In the current century, cardiovascular diseases are the leading causes of morbidity and mortality around the world, becoming a public health problem. According to the Pan American Health Organization, such diseases represented 31% of global deaths in 2016, and this situation is increasing in Brazil in 2021, until the month of November, estimated at 343,170 deaths according to the Brazilian Society of Cardiology^(1)^.

In most cases, when there is no improvement in the clinical condition through conventional treatment, a surgical approach is required, in which procedures are performed in order to reverse the clinical condition of the patient in question^(2)^.

In this sense, cardiac surgery is a procedure with relevant repercussions for the patient’s hemodynamic stability, becoming increasingly effective and safe^(3)^. Therefore, from the epidemiological perspective that the main cause of death in the next decade will be from heart disease, the application of new and advanced technologies during the procedure can be noted^(4)^.

Extracorporeal circulation (CPB), for example, consists of replacing, in a specific and limited time, the function of the heart and lungs during cardiac surgery^(5)^. In this way, the equipment enables blood oxygenation through a machine that acts on the propulsion and aspiration of blood, promoting oxygenation using a reservoir membrane and thus enabling the replacement of cardiac function and pumping of blood to the body. It is an essential mechanism for cardiac surgery, however it can cause several complications in the postoperative period^(6)^. This is because surgical intervention and the immediate postoperative period are periods in which the appearance of so-called common complications is observed, such as the patient’s hemodynamic instability^(7)^.

During cardiac surgery using CPB, it is important to identify a biomarker of circulatory failure, called “hyperlactatemia” and “lactic acidosis”, a fact that occurs when the body in anaerobic condition transforms pyruvate into lactate; this is due to a low oxygen debt, increasing the severity of tissue hypoperfusion^(8)^. In this sense, hyperlactatemia is commonly found in the postoperative period of cardiac surgery^(9)^.

For some authors, this disorder occurs because of the low supply of oxygen to tissues, which results in circulatory dysfunction. This, in turn, manifests as alterations in hemodynamic parameters - such as heart rate, blood pressure, decreased tissue perfusion - and changes in metabolic markers, such as lactate levels. That said, the literature shows that increased lactate in critically ill patients has been related to the presence of severe circulatory and various organ dysfunction^(10)^.

Much is known about the relationship between lactate and major postoperative complications. Therefore, the self-justification of this work lies in its contribution to knowledge and to the scientific community of bringing to light mapped data on the main variables related to this increase in lactate in cardiac surgeries with the use of CPB. In this way, it can contribute to the prevention of postoperative mortality and better prognoses for these patients. Likewise, it contributes to nursing science, given the importance of the perfusionist nurse in the use of CPB in cardiac surgeries, in providing nursing care throughout the preoperative, intraoperative, and postoperative process.

A prior search was carried out on the following international registry platforms: International Prospective Register of Systematic Reviews, Open Science Framework, The Cochrane Library, JBI Clinical Online Network of Evidence for Care and Therapeutics and Database of Abstracts of Reviews of Effects. No similar research to the theme of this study was found in them, reinforcing the importance of carrying it out.

Given the above, the question arises: What are the factors associated with increased lactate levels in the postoperative period of cardiac surgeries using cardiopulmonary bypass?

## OBJECTIVES

To map the factors associated with increased lactate levels in the postoperative period of cardiac surgery using extracorporeal circulation.

## METHODS

### Ethical aspects

Considering that the data included in this review study are in the public domain, this research was not submitted to the Research Ethics Committee.

### Study design

The present study is a scoping review that follows the recommendations of the Joanna Briggs Institute^(11)^ and the Preferred Reporting Items for Systematic Reviews and Meta-Analyses Extension for Scoping Review^(12)^ checklist. Its purpose is to map the main concepts in a given area of research, identify gaps that still exist in knowledge, as well as highlight the need to develop new future research. Given this, the main indications chosen for preparing the review based on the JBI guidelines were with the aim of identifying the types of evidence available in a given field; identify and analyze knowledge gaps; and to identify the main characteristics or factors related to a concept. This research was also registered on the Open Science Framework platform.

To plan this study, the steps of Arksey and O’Malley^(13)^ improved by Peters et al. (2020)^(14)^ were utilized: 1) Define and align the objective and research question; 2) Develop and align inclusion criteria with the objective and research question; 3) Describe the planned approach to evidence search, selection, data extraction and presentation of evidence; 4) Search for evidence; 5) Select the evidence; 6) Extract the data; 7) Analyze, present and summarize the results.

To formulate the research strategy, the mnemonic PCC (Population, Concept and Context) was used as indicated by JBI^(9)^, in which P - Adult patients undergoing cardiac surgery; C - Factors associated with increased postoperative lactate levels; C - Using extracorporeal circulation. Therefore, the following research question was created: “What are the factors associated with increased lactate levels in the postoperative period of cardiac surgeries using extracorporeal circulation?”.

### Study period and location

The collection was carried out from November 18 to 25, 2022, using the Periodical Portal of the Coordination for the Improvement of Higher Education Personnel, through remote access through the Federated Academic Community platform, through the Federal University of Rio Grande do Norte.

The descriptors were consulted in Health Sciences Descriptors (DeCS) and Medical Subject Headings (MeSH), aiming to adapt the searches to Portuguese and English. These were: “Thoracic Surgery”, “Thoracic Surgery”, “Cardiac Surgery”, “Lactic Acid”, “Lactic Acid”, “Lactate”, “Mortality”, “Mortality”, “Mortality Index”, “Postoperative Period” , “Postoperative Period”, “Cardiopulmonary Bypass”, “Extracorporeal Circulation”. To cross the descriptors, the Boolean operator “AND” was used. It is worth noting that the descriptor “hyperlactatemia” was not included in the syntax because it significantly restricted the search for studies. We opted for a more comprehensive search in the databases, including “Lactic Acid” as a descriptor and “hyperlactatemia” or “increased lactate” as an inclusion criterion in the selection of articles.

Ten data sources were used in this search: Scopus, PubMed Central, Web of Science, Science Direct, Scientific Electronic Library Online (SciELO), Cochrane Library, Latin American and Caribbean Literature in Health Sciences (LILACS), Wiley Online Library and Cumulative Index to Nursing and Allied Health Literature (CINAHL); and, for gray literature, the Catalog of Theses and Dissertations (CAPES). The search strategy was adapted according to the specificities of each source used, however, combinations between descriptors were preserved and time and language restriction filters were not added (Chart 1).

**Chart 1:** Search syntax for articles in the data sources, Natal, Rio Grande do Norte, Brazil, 2021

Source	Syntax
Scopus	ALL (thoracic AND surgery AND lactic AND acid) AND ALL (cardiopulmonary AND bypass AND postoperative AND period) AND ALL (mortality) AND (LIMIT-TO (OA, “all”))
PubMed PMC	(((Thoracic Surgery AND Lactic Acid)) AND (cardiopulmonary bypass and Postoperative period)) AND Mortality
Web of Science	((ALL=(Thoracic Surgery AND Lactic Acid)) AND ALL=(cardiopulmonary bypass AND Postoperative period)) AND ALL=(Mortality)
Science Direct	(Thoracic Surgery AND Lactic Acid) AND (cardiopulmonary bypass and Postoperative period) AND Mortality
Scientific Electronic Library Online	(Thoracic Surgery AND Lactic Acid) AND (cardiopulmonary bypass and Postoperative period) AND (Mortality)
Cochrane Library	Thoracic Surgery AND Lactic Acid in Title Abstract Keyword AND cardiopulmonary bypass and Postoperative period in Title Abstract Keyword AND Mortality in Title Abstract Keyword
Latin American and Caribbean Literature in Health Sciences	ALL (thoracic AND surgery AND lactic AND acid) AND ALL (cardiopulmonary AND bypass AND postoperative AND period) AND ALL (mortality) AND (LIMIT-TO (OA, “all”))
Wiley Online Library	“Thoracic Surgery AND Lactic Acid” anywhere and “cardiopulmonary bypass and Postoperative period” anywhere and “Mortality” anywhere
Cumulative Index to Nursing and Allied Health Literature	(Thoracic Surgery AND Lactic Acid) AND (cardiopulmonary bypass and Postoperative period) AND Mortality
Catalog of Theses and Dissertations	(Thoracic Surgery AND Lactic Acid) AND (cardiopulmonary bypass and Postoperative period) AND (Mortality)

### Inclusion and Exclusion Criteria

These were the inclusion criteria: scientific articles published online in full, dissertations, theses, guidelines, which addressed the relationship between increased lactate levels in cardiac surgeries using cardiopulmonary bypass in adult patients; be made available via remote access from the Federated Academic Community; not have time or language restrictions; answer the proposed research question. Abstracts, letters to the editor, opinion articles, studies that were off topic and duplicate records in the data source were excluded; pediatric and neonatal audiences.

### Study protocol

The data search, screening and inclusion of studies were carried out by two independent evaluators simultaneously and on different electronic devices. The divergences found between the reviewers during the selection process were resolved by them in meetings or by a third researcher consulted to read the material in full and perform the tiebreaker for the composition of the final sample. After discussion, a decision was made on inclusion or exclusion from the study. No software was used to manage references or exclude duplicates.

Furthermore, the PAGER methodology^(13)^ was used as a facilitator to improve the quality of reports, in order to establish greater methodological rigor to this review and provide greater clarity of the results of scoping reviews, offering a consistent approach to the analysis and report. Therefore, given the importance of this device, this review will formulate a picture of such a structure.

### Analysis of results

After selecting the studies and reading them in full, the data obtained from the records were placed in a Microsoft Excel spreadsheet created by the authors and were synthesized into charts in order to facilitate the understanding of the results by readers. The analysis of the studies occurred in a descriptive way.

## RESULTS

**Figure 1:**
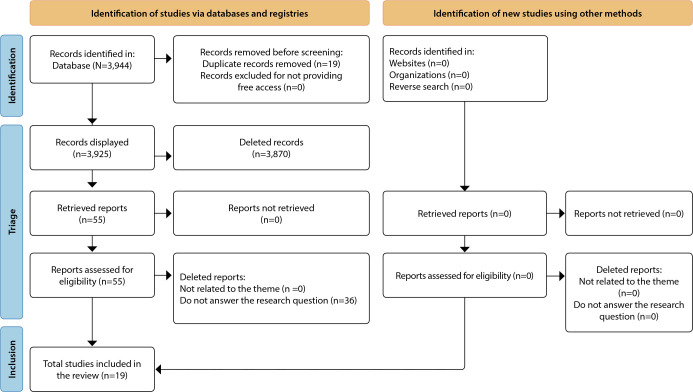
Search flowchart adapted from PRISMA-ScR, Natal, Rio Grande do Norte, Brazil, 2022

Figure 1 presents the process of identification, screening, eligibility, and inclusion of studies to select the sample of results.

*n - number.

In total, 3,944 scientific articles were found in the data sources, of which 247 were found in PubMed Central; 3,328, at CINAHL; 201, in Scopus; 17, at Wiley on Library; and 151, in Science Direct. No results were obtained in the Cochrane Library, Web of Science, LILACS, SciELO and CAPES databases. Of the total, 19 studies were excluded due to duplicates and then, after applying the inclusion and exclusion criteria, 55 studies were selected for full reading, among which 36 were excluded for not answering the research question, resulting in the composition of 19 articles in the final sample.

As for the year of publication, the studies ranged from 2006 to 2022. It was noticed that, from 2015 onwards, there was a significant increase in the number of research on lactate, highlighting the years 2015 and 2022, as they were those in that 80% of the selected studies were published. Furthermore, there was a prevalence of studies carried out in Germany, Saudi Arabia, and China, with two publications in each.

Retrospective and prospective observational studies corresponded to the predominant class in the sample, representing 12 articles, followed by randomized controlled and prospective randomized studies with 5 articles, as well as a cohort study and case-control study representing 1 article from each category. There was agreement regarding the types of cardiac surgery with the use of extracorporeal circulation, with valve replacement (mitral/aortic) and myocardial revascularization predominating in 100% of the selected articles, with CPB time varying between 110 and 148 minutes in 30% of articles; most articles reported that surgeries were elective.

Aiming to extend the rigor and increase the quality of this scoping review, Chart 2 summarizes the information that answers the research question and the main outcomes found in the findings.

**Chart 2:** Summary of studies included in the review, Natal, Rio Grande do Norte, Brazil, 2022

Year/ Country/ Reference	Type of cardiac surgery/ Average CPB time	Main factors associated with increased lactate	Outcome
2022/ China^(15)^	Heart valve surgery/ 140 min	Hypoperfusion, intraoperative sufentanil infusion rate, use of epinephrine	Inadequate intraoperative sufentanil infusion rate is an independent risk factor for acidosis.
2022/ Morocco^(16)^	Valvular surgery, coronary artery bypass grafting, combined procedure, adult congenital anomalies and aortic surgery/ 120 min	Hypoperfusion of body tissues and hypoxia cellular, duration of CPB, severe hemodilution	Blood lactate levels above 5 mmol/L at the end of CPB are associated with worse outcomes.
2022/ Turkey^(17)^	Myocardial revascularization/ Time not reported	Kidney injury, ischemic stroke, hyperglycemia, and blood transfusion	Longer CPB time was associated with higher lactate levels and increased mortality.
2020/ Spain^(18)^	Valve, coronary and aortic replacement surgeries/ 102 min	Hemodilution and CPB time	Reduction in lactatemia resulted in a decrease in morbidity and mortality in the intensive care unit.
2020/ Japan^(19)^	Valve surgeries, CABG, aortic surgeries/Time not reported	Age, preoperative history of HF, surgery time, CPB time, aortic clamping, and use of adrenaline	Early onset of a maximum arterial lactate concentration greater than 4.5 mmol/L was significantly associated with in-hospital mortality.
2019/ Poland^(20)^	Heart valve surgery/ 122 min	Tissue hypoperfusion	The blood lactate level measured one day after the operation was an independent predictor of death at a 30-day follow-up.
2018/ Germany^(21)^	Unspecified type of surgery/ 129 min	Tissue hypoxia, drug therapy, hypothermia	Excess base severely reduced on ICU admission is superior to lactate levels for predicting ICU mortality.
2018/ Germany^(22)^	Type of surgery not mentioned/5 to 10 min	CPB time	In patients who received CPB for two hours or more, venous lactate and partial pressure of carbon dioxide in the blood were higher than baseline values.
2017/ Saudi Arabia^(23)^	Myocardial revascularization / 100 min	Tissue anoxia caused by increased vasopressor support after surgery	Elevated lactic acid levels appear to be directly related to tissue anoxia caused by increased vasopressor support after surgery.
2017/ United Kingdom^(24)^	Mitral valve surgery/ Time not reported	CPB times, epinephrine use, preoperative atrial fibrillation, and preoperative heart failure	A high level of hyperlactatemia (7 mmol/L) was associated with mortality, but the majority of those who died did not have high levels upon admission to the ICU.
2016/ Canada^(25)^	Myocardial revascularization and/or valve surgery/ 130 min	Tissue hypoperfusion	Mortality is proposed to be secondary to a state of ongoing hypoperfusion.
2016/ United States^(26)^	Myocardial revascularization and valve surgery/Time not reported	Low ejection fraction, age, reoperation, diabetes, hypertension	No difference was found in the postoperative period in relation to lactate levels and its association with mortality.
2015/ Saudi Arabia^(27)^	Not reported in study	Hemofiltration during CPB	Hemofiltration during CPB leads to hemoconcentration, increased serum lactate and inotropic support.
2015/ China^(28)^	Lung bypass surgery/ 83 min	Administration of lactated Ringer’s solution, distributive shock, type B hyperlactatemia	There was no direct causal relationship between lactate index and mortality.
2015/ Netherlands^(29)^	Unspecified surgery/ 148 min	High dose of intraoperative dexamethasone	High doses of intraoperative dexamethasone increased postoperative lactate and glucose levels within the first 15 hours of ICU admission.
2013/ Brazil^(30)^	Not mentioned in the study	A threshold of 3 mmol/L within 6 hours of ICU admission and 2 mmol/L	After 6 hours of ICU admission, lactate level predicts postoperative complications such as 30-day all-cause mortality and severe morbidity after cardiac surgery in adult patients.
2011/ Australia^(31)^	Unreferenced type/ 50 to 100 min	Increase in lactate associated with CPB time	In patients who received CPB for two hours or more, venous lactate and PvCO_2_ were higher than baseline values.
2011/ India^(32)^	Myocardial revascularization and valve surgery/ > 60 min	CPB time	Pre-CPB myocardium, lactate value 2.9 mmol/l, myocardial pyruvate value of 0.07 mmol/l can predict post-CPB inotropic need with good sensitivity and specificity.
2006/ Sweden^(33)^	Myocardial revascularization/ 90.2 min	Age, use of vasoactive drugs, CPB time, aortic clamping time	In patients who met the criteria for hyperlactatemia, in-hospital mortality was 13.3%, compared with 2.2% for the entire cohort.

* *CPB - extracorporeal circulation.*

Therefore, Chart 3 shows the mapping of all risk factors mentioned throughout the studies and their relationship with the increase in lactate in the postoperative period of cardiac surgery with the use of extracorporeal circulation.

**Chart 3:** Prevalence of risk factors cited in studies, Natal, Rio Grande do Norte, Brazil, 2022

RISK FACTORS	CITATIONS IN STUDIES
Age	1
Sex	2
Comorbidities	3
Extracorporeal circulation time	9
Surgery time	2
tissue hypoperfusion	13
distributive shock	1
Hyperglycemia	1
Cardiac insufficiency	3
Lactate source lungs	1
Low splenic circulation	1
Low hemoglobin	1
Anoxia time	5
Accelerated glycolysis	2
Peripheral arterial disease	1
Vasoactive drugs	1
Corticosteroid use	6
Low ejection fraction	1
Kidney dysfunction	3
Use of the intra-aortic balloon	3
Blood transfusion	1
Hemodilution	3
Ineffective liver clearance	1
Ineffective cardioplegia	3
Hypothermia	2

In this sense, Chart 4 shows the PAGER structure according to the analysis of selected articles on the factors of increased lactate in the postoperative period of cardiac surgery with the use of extracorporeal circulation.

**Chart 4:** PAGER structure obtained from the analysis of selected articles, Natal, Rio Grande do Norte, Brazil, 2022

Standard	Advances	Gaps	Evidence for practice	Search recommendations
Hypoperfusion/Tissue hypoxia	The important role of perioperative tissue hypoxia in the development of postoperative complications	Some factors related to sample size, data and validity compromise the quality of the studies.	The study is relevant for helping and alerting the healthcare team about possible complications after heart surgery.	It is necessary to carry out additional studies in this line to guarantee the results of the data.
CPB time	Prolonged CPB time and other factors are associated with early and late postoperative hyperlactatemia.	Studies with small samples of participants, harmful to inferring results	The study contributes to the health team’s performance regarding postoperative results, depending on the patient’s physiological needs.	More research on this topic with larger sample sizes is needed.
Use of drugs	Inadequate intraoperative infusion of high-dose medications is an independent risk factor for lactic acidosis after cardiac surgery.	Few studies address the relationship between specific drugs and increased lactate.	Evaluates the risk-benefit of intraoperative drug use.	Studies with different drug therapy approaches are needed for a more comprehensive investigation.

* *CPB - extracorporeal circulation.*

## DISCUSSION

In the analysis of the results obtained in the selected studies, regarding the risk factors that answer the research question, it was possible to observe that, in the vast majority, the cause of increased lactate in cardiac surgery was related in a multifactorial manner, with the indication of more a factor in the same study. The most prevalent precursors to increased lactate were: tissue hypoperfusion, cited in 13 studies; CPB time, in 9 studies; and use of corticosteroids, in 6 studies.

Hyperlactatemia is usually identified in patients undergoing cardiac surgery procedures. When associated with the extracorporeal circulation device, it can cause metabolic changes that, with the cardiac biochemical processes involved in the surgery, alter the serum level of blood lactate^(34)^.

In this sense, the increase in lactate is understood as a biomarker of the response to stress, called a multifactorial process and not just reflections of tissue hypoperfusion. Thus, hyperlactatemia is classified into type A and type B. Type A hyperlactatemia is often associated with the postoperative period of cardiac surgery due to the emergence of metabolic acidosis, that is, it results from anaerobic metabolism evidenced by the low supply of oxygen required by the cellular metabolism, resulting in tissue hypoxia^(35)^.

Type B hyperlactatemia, however, results from extreme aerobic conditions such as stress and medication use, in addition to malnutrition, malignant diseases and metabolic errors. In this circumstance, the use of epinephrine in cardiac surgeries is mentioned, which contributes to the processes of glycolysis and gluconeogenesis and, in this way, promotes the increase in lactate with type B hyperlactatemia^(36)^.

Thus, other intraoperative and postoperative factors for variation in lactate values stand out in the literature: decreased blood flow, circulatory arrest, temperature, CPB time, oxygen supply, hematocrit level during and after CPB and cardioplegia solution., in addition to the system’s inflammatory responses. Therefore, it is clear that hyperlactatemia can result from both hypoxic and non-hypoxic causes^(37)^.

Within this context, studies discuss normal values for lactate levels and their estimated values, as there is an evident increase in these values during CPB; however, the blood lactate level is 0.5-2.2 mmol/L in physiological conditions, therefore a warning sign would be defined above 3 mmol/L during CPB, while other authors report a peak above 4 mmol/L as a better predictor of mortality^(38)^. This is in line with the findings of the present study, regarding the serum level of 4 mmol/L as a predictor of greater postoperative complications and higher mortality.

From this perspective, the study by Horak^(34)^ associates serum lactate concentration above 3 mmol/L in cardiac surgery with a greater risk of postoperative hospital morbidity and mortality, in which patients with hyperlactatemia were 3.85 times more likely to die. Furthermore, the incidence of hyperlactatemia in the cohort was 45.2%, with a mortality rate of approximately 14%, confirming the findings presented in relation to the proportionality of lactate and mortality.

So, when studies talk about extracorporeal circulation flow and its intraoperative management, it must be borne in mind that intraoperative patient management needs to have a multifaceted approach to provide adequate oxygen supply to the tissues during CPB, offering at least 280 ml/min/m^2^ and cardiac index of 2.4 L/min/m^2^. Flow and its management can be administered independently or according to the patient’s metabolic needs - for example, hemoglobin concentration, temperature, diuresis and vascular resistance - thus resulting in a wide variation in CPB conduction^(35)^.

In the same reasoning, in the search for preventive measures/actions and considering that CPB time is directly related to this increase in lactate, the best scenario to maintain lactate at physiological levels is to seek hemodynamic stability in the patient pre-operatively so that there is a shorter CPB time possible and less use of vasoactive drugs during the procedure^(38)^.

The perfusionist nurse is of fundamental importance for the progress of each stage of cardiac surgery, in addition to being responsible for providing humanized assistance to patients at all stages of hospitalization, helping them with their every need. In relation to CPB, the nurse can apply the Nursing Care Systematization (SAE) process in order to better conduct the procedure and avoid and/or reduce risks^(39)^.

### Study limitations

The limitations of this study include: the fact that not all factors have their origin in specific and in-depth studies; and the scarcity of clinical trials focused on the topic. Therefore, further studies aimed at factors associated with hyperlactatemia are needed.

### Contributions to the area

The present study can contribute to the prevention and promotion of health in the postoperative period of patients undergoing cardiac surgery using CPB, as it provides data based on scientific evidence on the main factors associated with elevated lactate.

## CONCLUSIONS

Among the findings, cardiopulmonary bypass time, tissue hypoperfusion and use of corticosteroids stand out as the main factors associated with increased lactate. Furthermore, in 95% of the selected studies, the increased mortality of surgery patients undergoing cardiopulmonary bypass was directly related to the increase in lactate. The value between 3 mmol/L and 4 mmol/L was referred to as outside the physiological normality, offering risk, in addition to that 4 mmol/L is considered the limit value for greater postoperative complications and higher mortality.

In conclusion, it is understood that discussing the causes of possible complications in cardiac surgery patients is important for preparing the team and preventing complications, in addition to ensuring quality recovery, with a positive prognosis and suitable for hospital rehabilitation. It is expected to arouse greater interest in the topic with greater scientific investment, for the development of more research in the area.

## References

[1] Siqueira SM, Braga GT, Martins SP, Ribeiro TC. (2022). Intervenções adotadas pela enfermagem frente às principais complicações no pós-operatório de cirurgias cardíacas com uso de circulação extracorpórea em adultos. REASE.

[2] Amorim TV, Salinema AM. (2015). Processo cirúrgico cardíaco e suas implicações no cuidado de enfermagem: revisão/reflexão. HU Rev.

[3] Caldeira C, Soares AJC. (2017). Perfil clínico e epidemiológico dos pacientes que realizaram cirurgia cardíaca no hospital sul fluminense - HUSF. Rev Saúde.

[4] Silva PL, Damasceno RF. (2020). Infecções hospitalares em pacientes submetidos à cirurgia cardíaca: uma revisão das incidências quanto aos fatores de risco pós-cirurgia. J Manag Prim Health Care.

[5] Matias ML, Reis VA. (2022). Benefícios da técnica de ultrafiltração com balanço zero (Z-BUF) durante a circulação extracorpórea em pacientes submetidos à cirurgia cardíaca. RECISATEC.

[6] Kakihara KS. (2018). Validação de um guia de boas práticas para o cuidado realizado pelo enfermeiro ao paciente em circulação extracorpórea.

[7] Borges MGB. (2017). Influência dos marcadores de hipoperfusão tecidual na força muscular periférica e capacidade funcional em pacientes no pós-operatório de cirurgia cardíaca.

[8] Hoshino Y, Kinoshita O, Ono M. (2018). The incidence, risk factors, and outcomes of hyperlactatemia after heart transplantation. Int Heart J.

[9] Horak ACP, Ferretti-Rebustini REL, Oliveira LB, Crespo JCL, Wilson AMMM, Oliveira JC (2022). Hyperlactatemia and worse outcomes in patients undergoing cardiac surgery: a retrospective cohort study. Rev Paul Enferm.

[10] Matos SINS. (2021). Nível de lactato como indicador prognóstico de mortalidade e morbilidade hospitalar.

[11] Peters MDJ, Godfrey C, McInerney P, Munn Z, Tricco AC, Khalil H., Aromataris E, Munn Z (2020). Joanna Briggs Institute Reviewer´s Manual, JBI.

[12] Tricco AC, Lillie E, Zarin W, O'Brien KK, Colquhoun H, Levac D (2018). PRISMA Extension for Scoping Reviews (PRISMA-ScR): checklist and explanation. Ann Intern Med.

[13] Arksey H, O'Malley L. (2005). Scoping studies: towards a methodological framework. Int J Soc Res Methodol.

[14] Peters MD, Marnie C, Tricco AC, Pollock D, Munn Z, Alexander L (2020). Updated methodological guidance for the conduct of scoping reviews. JBI Evid Synth.

[15] Zhan YF, Shi Q, Pan YC, Zheng BS, Ge YP, Luo TG (2022). Sufentanil: a risk factor for lactic acidosis in patients after heart valve surgery. J Cardiothorac Surg.

[16] Seghrouchni A, Atmani N, Moutakiallah Y, Belmekki A, Bekkali YE, Houssa MA. (2021). Does severe hyperlactatemia during cardiopulmonary bypass predict a worse outcome?. Ann Med Surg.

[17] Özmen R, Bozguney M, Tekin AI, Eroglu T, Tuncay A. (2022). Impact of Single versus Double Clamp Technique on Blood Lactate Levels and Postoperative Complications in Coronary Artery Bypass Surgery. Braz J Cardiovasc Surg.

[18] Camacho CG, Paz AJ, Franco CL, Hervás MJ, Otero AM. (2020). Continuous ultrafiltration during extracorporeal circulation and its effect on lactatemia: a randomized controlled trial. PLoS One.

[19] Ezaka M, Tsukamoto J, Matsuo K, Kin N, Yamaoka K. (2020). Hyperlactatemia of dialysis-dependent patients after cardiac surgery impacts on in-hospital mortality: a two-center retrospective study. JA Clin Rep.

[20] Duchnowski P, Hryniewiecki T, Kuśmierczyk M, Szymański P. (2019). The usefulness of perioperative lactate blood levels in patients undergoing heart valve surgery. Kardiochir Torakochirurgia Pol.

[21] Piot J, Hébrard A, Durand M, Payen JF, Albaladejo P. (2019). An elevated respiratory quotient predicts complications after cardiac surgery under extracorporeal circulation: an observational pilot study. J Clin Monit Comput.

[22] Zante B, Reichenspurner H, Kubik M, Kluge S, Schefold JC, Pfortmueller CA. (2018). Base excess is superior to lactate-levels in prediction of ICU mortality after cardiac surgery. PLoS One.

[23] Nazer RI, Alburikan KA. (2017). Metformin is not associated with lactic acidosis in patients with diabetes undergoing coronary artery bypass graft surgery: a case control study. BMC Pharmacol Toxicol.

[24] Yusuff HO, Zochios V. (2018). Lactic Acidosis and Mitral Valve Surgery: defining the relationship. J Cardiothorac Vasc Anesth.

[25] Mak NT, Igbal S, Varennes B, Khwaja K. (2016). Outcomes of post-cardiac surgery patients with persistent hyperlactatemia in the intensive care unit: a matched cohort study. J Cardiothorac Surg.

[26] Andersen LW, Holmberg MJ, Berg KM, Chase M, Cocchi MN (2016). Thiamine as an adjunctive therapy in cardiac surgery: a randomized, double-blind, placebo-controlled, phase II trial. Crit Care.

[27] Soliman R, Fouad E, Belghith M, Abdelmageed T. (2016). Conventional hemofiltration during cardiopulmonary bypass increases the serum lactate level in adult cardiac surgery. Ann Card Anaesth.

[28] Zhang Z, Ni H. (2015). Normalized lactate load is associated with development of acute kidney injury in patients who underwent cardiopulmonary bypass surgery. PLoS One.

[29] Ottens TH, Nijsten MW, Hofland J, Dieleman JM, Hoekstra M, Dijk D (2015). Effect of high-dose dexamethasone on perioperative lactate levels and glucose control: a randomized controlled trial. Crit Care.

[30] Hajjar LA, Almeida JP, Fukushima JT, Rhodes A, Vincent JL, Osawa EA (2013). High lactate levels are predictors of major complications after cardiac surgery. J Thorac Cardiovasc Surg.

[31] Dong MF, Ma ZS, Wang JT, Chai SD, Tang PZ, Wang LX. (2012). Impact of peripherally established cardiopulmonary bypass on regional and systemic blood lactate levels. Heart Lung Circ.

[32] Kapoor P, Mandal B, Chowdhury U, Singh S, Kiran U. (2011). Changes in myocardial lactate, pyruvate and lactate-pyruvate ratio during cardiopulmonary bypass for elective adult cardiac surgery: early indicator of morbidity. J Anaesthesiol Clin Pharmacol.

[33] Svenmarker S, Häggmark S, Ostman M. (2006). What is a normal lactate level during cardiopulmonary bypass?. Scand Cardiovasc J.

[34] Horak AC, Ferriti-Rebustini RE, Oliveira LB, Crespo JC, Wilson AM, Oliveira JC (2022). Hyperlactatemia and worse outcomes in patients undergoing cardiac surgery: a retrospective cohort study. Rev Paul Enferm.

[35] Stephens EH, Epting CL, Backer CL, Wald EL. (2020). Hyperlactatemia: an update on postoperative lactate. World J Pediatr Congenit Heart Surg.

[36] Cotter EK, Kidd B, Flynn BC. (2020). Elevation of intraoperative lactate levels during cardiac surgery: is there power in this prognostication?. J Cardiothorac Vasc Anesth.

[37] Clingan S, Reagor J, Lombardi J. (2019). Retrospective analysis of cardiac index and lactate production on cardiopulmonary bypass for a congenital cardiac patient population. Perfusion.

[38] Algarni KD. (2020). The effect of hyperlactatemia timing on the outcomes after cardiac surgery. J Thorac Cardiovasc Surg.

[39] Silva IN, Guedes PF, Nunes NS, Freitas VL. (2022). The attributions of the perfusion nurse: extracorporeal circulation (ECC). Res Soc Dev.

